# Exosomes in cancer: small particle, big player

**DOI:** 10.1186/s13045-015-0181-x

**Published:** 2015-07-10

**Authors:** Xu Zhang, Xiao Yuan, Hui Shi, Lijun Wu, Hui Qian, Wenrong Xu

**Affiliations:** Jiangsu Key Laboratory of Medical Science and Laboratory Medicine, School of Medicine, Jiangsu University, 301 Xuefu Road, Zhenjiang, Jiangsu 212013 China

**Keywords:** Exosomes, Intercellular communication, Cancer, Biomarker, Target

## Abstract

Exosomes have emerged as a novel mode of intercellular communication. Exosomes can shuttle bioactive molecules including proteins, DNA, mRNA, as well as non-coding RNAs from one cell to another, leading to the exchange of genetic information and reprogramming of the recipient cells. Increasing evidence suggests that tumor cells release excessive amount of exosomes, which may influence tumor initiation, growth, progression, metastasis, and drug resistance. In addition, exosomes transfer message from tumor cells to immune cells and stromal cells, contributing to the escape from immune surveillance and the formation of tumor niche. In this review, we highlight the recent advances in the biology of exosomes as cancer communicasomes. We review the multifaceted roles of exosomes, the small secreted particles, in communicating with other cells within tumor microenvironment. Given that exosomes are cell type specific, stable, and accessible from body fluids, exosomes may provide promising biomarkers for cancer diagnosis and represent new targets for cancer therapy.

## Introduction

Exosomes are small, lipid bilayer membrane vesicles of endocytic origin. Exosomes can be defined by several common characteristics, including size (50–100 nm in diameter), density (1.13–1.19 g/ml), morphology (“cup” or “dish” shaped in transmission electron microscopy), and certain enriched protein markers (tetraspanins, TSG101, Hsp70). Initially discovered as the garbage bags for removal of unwanted material from cells, the role of exosomes in immune response is gradually recognized as they function in antigen presentation. More recently, the researchers reveal that exosomes contain proteins and nucleic acids that are functional when transferred into recipient cells. Exosomes have been shown to act as shuttles between cells by transmitting signals (referred to as communicasomes). In this review, we highlight the recent advances in the roles of exosomes in cancer with an emphasis on the potential of exosomes as diagnosis biomarker and therapy target.

### Biogenesis, release, and uptake of exosomes

**Fig. 1:**
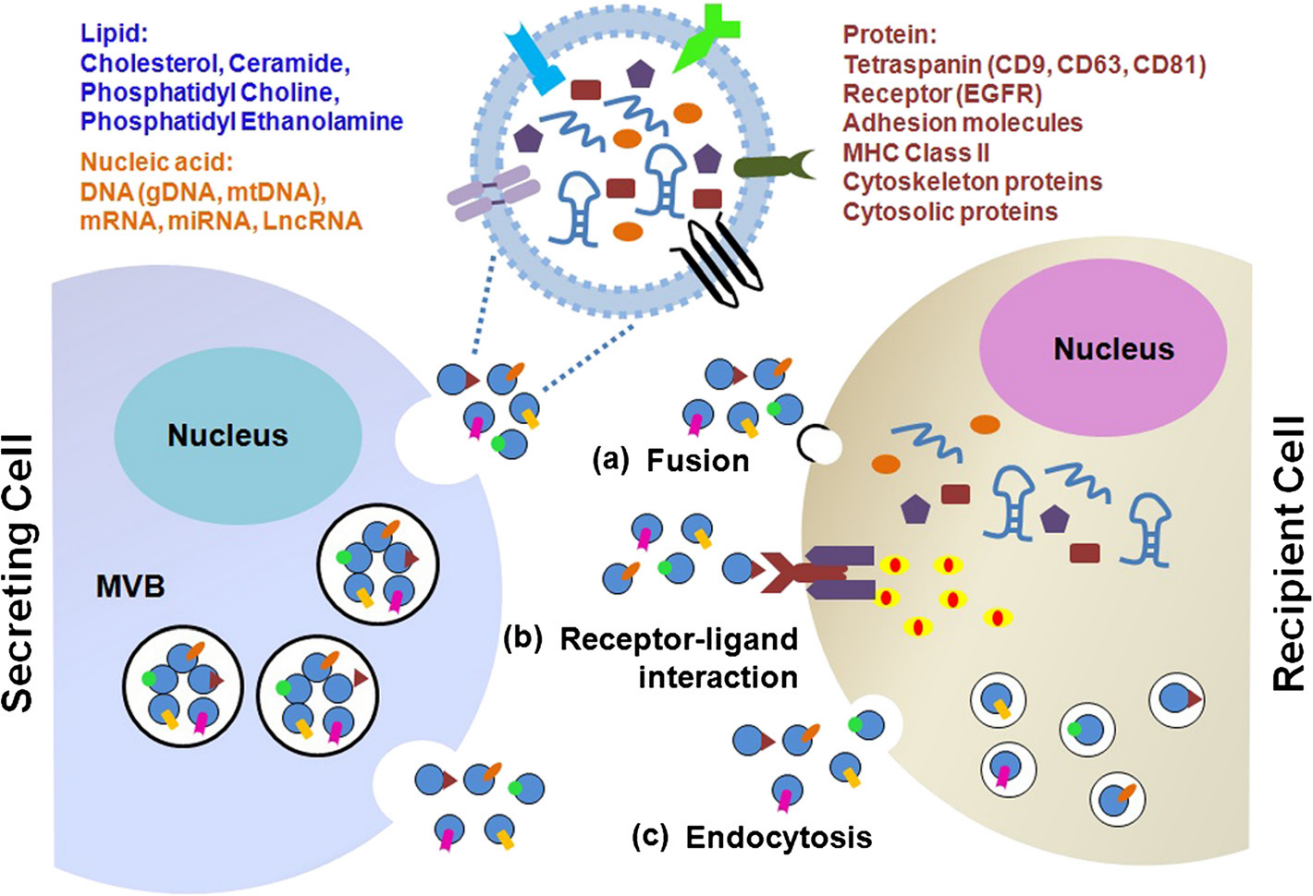
Biogenesis, release, structure, and uptake of exosomes. Exosomes are produced from the multivesicular bodies (MVBs) (also known as late endosomes). The membrane of the MVBs bulges inward to form exosomes. During this process, proteins (e.g., receptor, cytoplasmic proteins, tetraspanin), nucleic acids (e.g., DNA, mRNA, miRNA), and lipids (e.g., cholesterol, ceramide) are packed into exosomes in a cell type-dependent manner. MVBs fuse with the cellular membrane to release exosomes into the extracellular space. Several mechanisms have been suggested to mediate the uptake of exosomes, including **a** exosome fusion with the cellular membrane of the recipient cell, leading to the release of the exosomal cargo into the cytoplasm, **b** juxtracrine signaling through receptor-ligand interactions, **c** and endocytosis by phagocytosis

Exosome formation is a fine-tuned process which includes four stages: initiation, endocytosis, multivesicular bodies (MVBs) formation, and exosome secretion [1]. Multivesicular bodies (MVBs) are endocytic structures formed by the budding of an endosomal membrane into the lumen of the compartment. After vesicular accumulation, the MVBs are either sorted for cargo degradation in the lysosome or released into the extracellular space as exosomes by fusing with the plasma membrane (Fig. 1). The mechanisms underlying the sorting of cargo into the intraluminal vesicles (ILVs) are not yet fully elucidated. Both endosomal sorting complex required for transport (ESCRT)-dependent and independent signals have been suggested to determine the sorting of exosomes [2]. The formation of exosomes has been shown to be controlled by the syndecan heparan sulfate proteoglycans and their cytoplasmic adaptor syntenin [3].

The Rab guanosine triphosphatases (GTPases) have been found to critically regulate exosome secretion. Ostrowski et al. have identified that Rab27a/b affects the size and localization of MVBs [4]. Hsu et al. suggest that Rab3 regulates MVBs docking to tethering at the plasma membrane [5]. The accumulation of intracellular Ca^2+^ results in increased exosome secretion [6]. In addition, intracellular and intercellular pH has been shown to affect exosome release. When the microenvironmental pH is low, exosome secretion and uptake by recipient cells increases [7]. There is evidence that oncogenes and tumor suppressors regulate exosome secretion in cancer [8]. Yu et al. demonstrate that p53-regulated protein tumor suppressor-activated pathway 6 (TSAP6) induces exosome secretion under stressed conditions [9, 10]. Heparanase is an enzyme with elevated level in cancer. Overexpression of heparanase promotes exosome secretion [11]. Intriguingly, exosomes from normal mammary epithelial cells inhibit exosome secretion by breast cancer cells, implicating a feedback control to maintain dynamic equilibrium [12].

Exosomes transfer information to the target cells through three main ways: (1) receptor-ligand interaction; (2) direct fusion with plasma membrane; (3) endocytosis by phagocytosis (Fig. 1). Although the specific receptors that mediate the uptake of exosomes have not been found, there are several proteins that may act as potential receptors for exosome uptake, such as Tim1/4 for B cells [13] and ICAM-1 for APCs [14]. The uptake of exosomes by direct plasma membrane fusion mode has not been well studied. Melanoma cells could take up exosomes by fusion and low pH facilitates this process [15]. Phagocytosis is an efficient way of exosome uptake. Phagocytic cells have a greater uptake of exosomes than non-phagocytic cells [16]. The uptake of exosomes by recipient cells is energy dependent [17]. Heparan sulfate proteoglycans (HSPGs) function as internalizing receptors of cancer cell-derived exosomes. Enzymatic depletion of cell-surface HSPG or pharmacological inhibition of endogenous proteoglycan biosynthesis significantly attenuates exosome uptake [18].

### Structure and contents of exosomes

Exosomes consist of a lipid bilayer membrane surrounding a small cytosol (Fig. 1). The structured lipids not only mold the exosomes but are also involved in exosome function. In addition to lipids, nucleic acids and proteins have also been detected in exosomes. Thakur et al*.* demonstrate that double-stranded DNA is present in exosomes from cancer cells and reflects the mutational status of the originated cells [19]. Valadi et al. demonstrate that exosomes contain mRNA and miRNA [20]. Exosome-carried RNA can shuttle between cells and thus is called “exosomal shuttle RNA” (esRNA). The protein composition of tumor cell-derived exosomes has been well characterized for a number of cancers by using different proteomic methods. The most common proteins, mRNA, and miRNAs found in exosomes have been deposited in ExoCarta (www.exocarta.org). To date, 4563 proteins, 1639 mRNAs, and 764 miRNAs have been identified in exosomes from different species and tissues by independent examinations. The exosomal contents vary between different physiological and pathological conditions and original cell types. Moreover, the composition of exosomes can be distinct from the originated cells due to the selective sorting of the cargo into exosomes.

### Isolation, detection, and analysis of exosomes

Exosomes have been isolated and characterized from distinct cells under normal and stressed conditions. At present, the most commonly used methods for exosome isolation include ultracentrifugation, combined with sucrose gradient, and the immune-bead isolation (e.g., magnetic activated cell sorting; MACS). There are many commercial kits available for the extraction of exosomes. Transmission electron microscopy (TEM), Western blot, and FACS are frequently used to characterize the isolated exosomes based on their biochemical properties (e.g., morphology, size, exosomal markers). There is a lack of the accurate method to determine the concentration of exosomes. The researchers have to rely on inaccurate measurements of protein concentration or nanoparticle tracking analysis. Quantitative RT-PCR, nucleic acid sequencing, Western blot, or ELISA are used for exosome RNA and protein identification. The International Society for Extracellular Vesicles (ISEV) has recently released minimal experimental requirements for definition of extracellular vesicles and their functions [21].

### Roles of exosomes in cancer

**Fig. 2:**
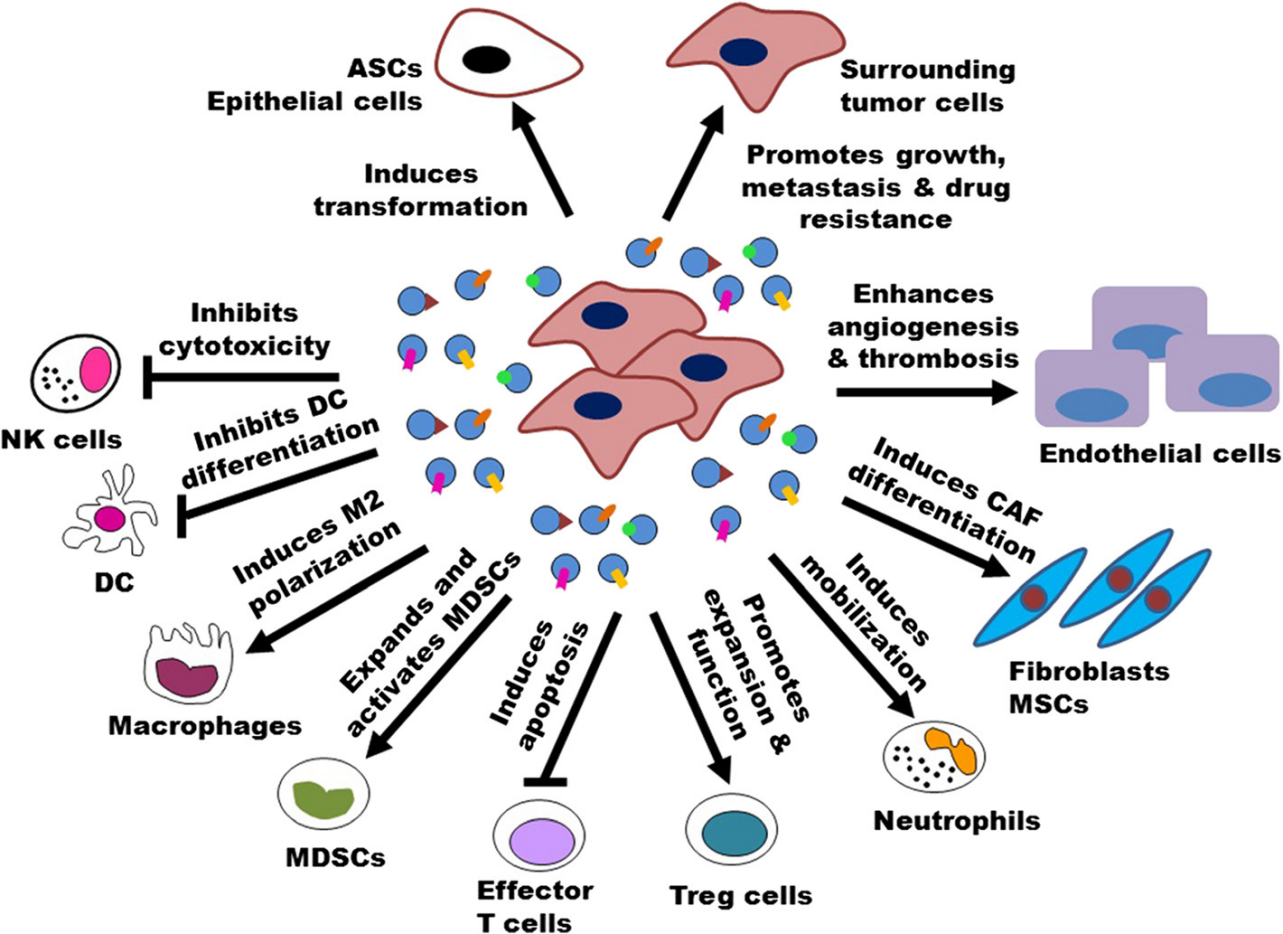
Roles of exosomes in cancer. Exosomes are critically involved in tumor initiation, growth, progression, metastasis, and drug resistance by transferring oncogenic proteins and nucleic acids. Tumor-derived exosomes can activate endothelial cells to support tumor angiogenesis and thrombosis. Tumor-derived exosomes can convert fibroblasts and MSCs into myofibroblasts to facilitate tumor angiogenesis and metastasis. Tumor-derived exosomes contribute to create an immunosuppressive microenvironment by inducing apoptosis and impairing the function of effector T cells and NK cells, inhibiting DC differentiation, expanding MDSCs, as well as promoting Treg cell activity. Tumor-derived exosomes can mobilize neutrophils and skew M2 polarization of macrophages to promote tumor progression. Moreover, tumor-derived exosomes can help tumor cells develop drug resistance by transferring multidrug-resistant proteins and miRNAs, exporting tumoricidal drugs, and neutralizing antibody-based drugs. In turn, exosomes from activated T cells, macrophages, and stromal cells can promote tumor metastasis and drug resistance

Accumulating evidence indicates that exosomes play important roles in cancer. Exosomes transfer oncogenic proteins and nucleic acids to modulate the activity of recipient cells and play decisive roles in tumorigenesis, growth, progression, metastasis, and drug resistance (Fig. 2). Exosomes can act on various recipient cells. The uptake of exosomes may induce a persistent and efficient modulation of recipient cells. In this section, we will discuss about the roles of exosomes in cancer and the molecular mechanisms (Table 1).

**Table 1:** Overview on the function of exosomes in cancer

Exosomal cargo	Secreting cell	Recipient cell	Function	Reference
EGFRvIII	Glioblastoma cells	Glioblastoma cells	Promotes tumor cell growth	[26]
Angiogenin, IL-8, VEGF	Glioblastoma cells	Endothelial cells	Promotes tube formation	[75]
∆Np73	Colon cancer cells	Colon cancer cells	Promotes tumor cell proliferation and therapy resistance	[27]
KRAS	Colon cancer cells (mutant KRAS)	Colon cancer cells (wild-type KRAS)	Enhances tumor cell growth	[97]
MET	Melanoma cells (highly metastatic)	Bone marrow progenitor cells	Promotes tumor growth and metastasis	[39]
HIF-1α	Nasopharyngeal carcinoma (NPC) cells (EBV-positive)	NPC cells (EBV-negative)	Promotes tumor cell migration and invasion	[37]
αvβ6 Integrin	Prostate cancer cells	Prostate cancer cells	Promotes tumor cell migration	[98]
Survivin	Cervical cancer cells	Cervical cancer cells	Inhibits genotoxic stress-induced apoptosis and promotes cell proliferation	[25, 99]
Wnt5a	Macrophages	Breast cancer cells	Enhances tumor cell invasion	[100]
Wnt3a	Diffuse large B-cell lymphoma side population (SP) cells	Neighboring non-SP cells	Modulates SP–non-SP transition and promotes tumor progression	[24]
FasL	Activated CD8+ T cells	Melanoma cells, lung cancer cells	Induces MMP9 expression and promotes lung metastasis	[43]
IL-6, CCL2, fibronectin	Multiple myeloma (MM) BM-MSCs	MM cells	Promotes tumor cell growth	[29]
Hsp72	Murine thymoma, mammary carcinoma, colon carcinoma cells	MDSCs	Induces immunosuppression and enhances tumor growth	[63]
TF	Squamous cells, colon cancer cells	Endothelial cells	Promotes coagulation	[71]
CD39, CD73	Bladder, colorectal, prostate, breast cancer cells	T cells	Induces adenosine production and inhibits T cell activation	[101]
TGF-β	Mesothelioma, prostate, bladder, colorectal, breast cancer cells	Fibroblasts	Induces myofibroblast differentiation and promotes tumor angiogenesis and growth	[66, 67]
TGF-β	Prostate cancer, gastric cancer	MSCs	Induces myofibroblast differentiation and promotes angiogenesis and invasiveness	[68, 102]
TGF-β	Pleural effusions of mesothelioma patients	NK cells, CD8+ T cells	Downregulates NKG2D expression and impairs cell killing activity	[103]
MICA*008	Cervical cancer cells	NK cells	Decreases NKG2D expression and reduces NK cytotoxicity	[104]
TGF-β, PGE2	Murine mammary adenocarcinoma cells	Bone marrow myeloid cells (CD11b+Ly6G+)	Induces MDSCs accumulation and immunosuppression	[61]
CCL20	Nasopharyngeal carcinoma cells	Regulatory T cells	Recruits and induces Treg conversion	[59]
KIT	Mast cells	Lung cancer cells	Accelerates cell proliferation	[105]
KIT	Gastrointestinal stromal tumor (GIST) cells	Progenitor smooth muscle cells	Increases tumor invasiveness	[40]
Wnt11	Fibroblasts	Breast cancer cells	Promotes tumor metastasis	[42]
MIF	Pancreatic cancer cells	Liver Kupffer cells	Promotes metastasis	[47]
Hsp70	Renal cancer cells (murine Renca cell line)	MDSCs	Induces MDSCs activation and enhances tumor growth	[106]
Adrenomedullin	Pancreatic cancer cells	Adipocytes	Promotes lipolysis	[107]
S1P, CCL20, PGE2	Enteropathogenic bacteria-stimulated intestinal epithelial cells	Th17 cells	Promotes the development of colon cancer	[108]
miR-9	Lung cancer, melanoma, pancreatic cancer, glioblastoma, colorectal cancer cells	Endothelial cells	Induces tumor angiogenesis	[109]
miR-125b, 130b, 155	Prostate cancer (PC) cells	PC patient adipose-derived stem cells (pASCs)	Induces neoplastic transformation	[22]
miR-135b	Multiple myeloma cells (under chronic hypoxia condition)	Endothelial cells	Enhances endothelial tube formation	[36]
miR-10b	Metastatic breast cancer cells	Mammary epithelial cells	Promotes cell migration	[110]
miR-92a	Chronic myeloid leukemia (CML) cells	Endothelial cells	Promotes cell migration and tube formation	[35]
miR-210	CML cells (under hypoxia condition)	Endothelial cells	Promotes angiogenic activity	[34]
miR-223	IL-4-activated macrophages	Breast cancer cells	Promotes cell invasion	[44]
miR-222	Drug-resistant breast cancer cells	Drug-sensitive breast cancer cells	Transmits chemoresistance	[111]
miR-584, 517c, 378	Hepatocellular carcinoma (HCC) cells	HCC cells	Promotes HCC cell growth and metastasis	[112]
miR-21, 29a	Lung cancer cells	Macrophages	Promotes tumor metastasis	[46]
miR-105	Metastatic breast cancer cells	Endothelial cells	Destroys tight junction, induces vascular permeability, and promotes metastasis	[33]
Pre-miRNAs, RISC-loading complex	Breast cancer cells	Non-tumorigenic epithelial cells	Induces cell transformation	[23]
miR-24-3p, 891a, 106a-5p, 20a-5p, 1908	Nasopharyngeal carcinoma	T cells	Promotes T cell dysfunction and tumor progression	[60]
miR-221, 222	Gastric cancer tissue derived MSCs	Gastric cancer cells	Enhances tumor cell migration	[60]
miR-122	Breast cancer cells	Lung fibroblasts, brain astrocytes, and neurons	Reprograms systemic energy metabolism and facilitates metastasis	[113]
miR-23b	Bladder cancer cells (cellular disposal by exosome release)	None	Acquires metastatic potential	[38]
miR-503	Endothelial cells	Breast cancer cells	Impairs tumor cell growth	[114]
miR-140	Preadipocytes	Ductal carcinoma in situ (DCIS) cells	Enhances tumorigenesis	[115]
miR-127, 197, 222, 223	Bone marrow stromal cells	Breast cancer cells	Decreases cell proliferation and induces cell quiescence	[116]
TUC339	Hepatocellular carcinoma (HCC) cells	HCC cells	Promotes tumor cell growth and inhibits cell adhesion	[81]
Linc-ROR	HCC cells	HCC cells	Reduces chemotherapy sensitivity	[82]

#### Tumorigenesis

Normal cells are transformed into cancer cells in the process of tumorigenesis. Exosomes from malignant cells have shown the potential to induce normal cell transformation. For instance, prostate cancer cell-derived exosomes could induce neoplastic transformation of adipose-derived stem cells (ASCs) [22], which is associated with trafficking of oncogenic proteins (Ras superfamily of GTPases), mRNA (K-ras and H-ras), as well as miRNAs (miR-125b, miR-130b, and miR-155) by exosomes. In addition, Melo et al. suggest that breast cancer cell-derived exosomes contain precursor microRNAs (pre-miRNAs) associated with RNA-induced silencing complex (RISC)-loading complex proteins, which could induce a rapid and efficient silencing of mRNAs in nontumorigenic epithelial cells, resulting in transcriptome reprogramming and oncogenic transformation [23]. They further demonstrate that the exosomes from serum specimen from breast cancer patients but not those from healthy donors induce tumor formation in mice when co-injected with the nontumorigenic epithelial cells, suggesting a potential mechanism for exosome in tumorigenesis. Cancer is composed of heterogeneous cell populations. Side population (SP) cells are a sub-population of cells that exhibit stem cell-like characteristics and can be isolated in cancer by adapting the Hoechst33342 staining method. Koch et al*.* demonstrate that in diffuse large B-cell lymphoma, side population cells could export Wnt3a via exosomes to neighboring cells, thus modulating SP-non-SP transitions and maintaining population equilibrium [24]. Altogether, these findings indicate that exosomes may contribute to tumor development and uncontrolled tumor progression by acting as a mediator in the transformation of normal cells to malignant cells and a modulator for the balance between cancer stem cells (CSCs) and non-CSCs.

#### Tumor growth

The promoting effects of exosomes from distinct sources on tumor cell proliferation have been widely reported. Cancer cells uptake exosomes that contain survivin, an anti-apoptotic protein, to protect them from genotoxic stress-induced cell death [25]. Exosomes from serum of glioblastoma patients contain EGFRvIII mRNA, which stimulate the proliferation of human glioma cells through a self-promoting way [26]. Colon cancer cell-derived exosomes are enriched in ΔNp73 mRNA. The proliferation potential of target cells is greatly enhanced by incubation with ΔNp73-containing exosomes [27]. The interaction between tumor stromal cells and tumor cells also efficiently promote tumor growth. Exosomes from chronic myelogenous leukemia (CML) cells stimulate bone marrow stromal cells to produce IL-8, which in turn promote the growth of leukemia cells [28]. Bone marrow mesenchymal stromal cells (BM-MSCs) from multiple myeloma (MM) patients release exosomes that express increased levels of oncogenic proteins, cytokines, and adhesion molecules to facilitate the growth of MM cells [29]. Thus, exosomes from tumor cells and microenvironment could act coordinately to promote tumor growth.

#### Tumor angiogenesis

The formation of new blood vessels is required for tumor growth and progression. Proteomic analysis has revealed that abundant angiogenic factors are present in malignant mesothelioma-derived exosomes [30]. Exosome uptake induces upregulation of angiogenesis-related genes and results in enhanced endothelial cell proliferation, migration, and sprouting [31]. Exosomes derived from hypoxic glioblastoma cells are more potent to induce angiogenesis [32]. Exosomes from metastatic breast cancer cells contain miR-105. Exosome-mediated transfer of miR-105 degrades ZO-1 protein, disturbs tight junctions, and induces vascular permeability in distant organs [33]. Exosomal miR-92a from K562 leukemia cells targets integrin α5 to enhance endothelial cell migration and tube formation [34]. MiR-210 is significantly enriched in exosomes from hypoxic K562 cells, which promotes the angiogenic activity of endothelial cells [35]. Multiple myeloma cells grown under hypoxic condition produce more exosomes containing miR-135b, which directly suppresses FIH-1, an inhibitor of HIF-1, to enhance endothelial tube formation in endothelial cells [36]. Exosomes are critically involved in tumor angiogenesis by directly delivering angiogenic proteins into endothelial cells or modulating the angiogenic function of endothelial cells by exosomal miRNAs.

#### Tumor metastasis

Exosomes contribute to tumor metastasis by enhancing tumor cell migration and invasion, establishing pre-metastatic niche, and remodeling the extracellular matrix. EBV-positive nasopharyngeal carcinoma (NPC) cell-derived exosomes contain HIF-1α, which increases migration and invasiveness of EBV-negative NPC cells [37]. Metastatic cancer cells secrete increased level of miRNA with tumor-suppressor function, which may suggest another mechanism for the role of exosomes in metastasis [38]. The formation of pre-metastatic niche is a prerequisite for tumor metastasis. Exosomes from highly metastatic melanoma enhance the metastatic ability of primary tumors by converting bone marrow progenitor cells to a pro-vasculogenic and pre-metastatic phenotype via the MET receptor [39]. Gastrointestinal stromal tumor cells release exosomes containing protein tyrosine kinase to convert progenitor smooth muscle cells to a pre-metastatic phenotype [40]. Suetsugu et al. show that highly metastatic breast cancer cells can transfer their own exosomes to other cancer cells and normal lung tissue cells in vitro and in vivo by using fluorescent protein imaging method [41], which provides direct evidence for the involvement of exosomes from highly metastatic cancer cells in educating stromal cells. Luga and colleagues have shown that exosomes produced by stromal cells are taken up by breast cancer cells and are then loaded with Wnt11, which is associated with stimulation of the invasiveness and metastasis of the breast cancer cells [42]. Exosomes from activated CD8+ T cells promote cancer cell invasion and lung metastasis via the Fas/FasL pathway [43], which adds another layer of mechanism for the role of tumor-infiltrating lymphocytes in cancer metastasis. Exosome-mediated transfer of oncogenic microRNAs into cancer cells is associated with enhanced metastatic potential. IL-4-activated macrophage-derived exosomes transfer miR-223 to co-cultivated breast cancer cells, leading to increase of cell invasion [44]. Exosome-mediated delivery of miR-221/222 from MSCs to gastric cancer cells greatly enhances gastric cancer cell migration [45]. Fabbri et al. suggest that miRNAs in tumor-secreted exosomes can directly bind toll-like receptor (TLR) in immune cells to promote tumor metastasis [46]. Recently, Costa-Silva and colleagues demonstrate that MIF-containing exosomes from pancreatic ductal adenocarcinoma (PDAC) cells induce TGF-β production in liver Kupffer cells, which in turn upregulates fibronectin (FN) expression by hepatic stellate cells and enhances recruitment of bone marrow-derived cells, finally leading to the formation of liver pre-metastatic niche [47], suggesting a complicated network that involves cancer cells, stromal cells, and immune cells in exosome-initiated pre-metastatic niche formation. Intriguingly, Zomer et al. use the Cre-LoxP system to visualize extracellular vesicle (EV) exchange between tumor cells in living mice [48]. They show that the less malignant tumor cells that take up EVs released by malignant tumor cells display enhanced migratory behavior and metastatic capacity, indicating that the metastatic behavior can be phenocopied through extracellular vesicle exchange. Taken together, these findings reveal that the intercellular communication mediated by exosomes may be an important mechanism for tumor metastasis.

#### Tumor drug resistance

Exosomes contribute to the development of therapy resistance in tumor cells through a variety of mechanisms. Tumor-derived exosomes can transfer multi-drug resistance (MDR)-associated proteins and miRNAs to target cells [49, 50]. In addition, exosomes participate in the process of tumor resistance by mediating drug efflux. The drugs and their metabolites can be encapsulated and exported by exosomes [51, 52]. Melanosomal sequestration of cytotoxic drugs contributes to the intractability of malignant melanomas [53]. Moreover, exosomes may counteract the effect of antibody drugs by modulating their binding to tumor cells. Lymphoma exosomes carry CD20, which bind therapeutic anti-CD20 antibodies and protect target cells from antibody attack [54]. Exosomes from HER2-overexpressing breast cancer cells express active HER2 and can bind to the HER2 antibody trastuzumab to inhibit its activity [55]. Exosomes secreted by stromal cells also contribute to tumor drug resistance. BM-MSC-derived exosomes induce multiple myeloma cells resistant to bortezomib through the activation of several survival relevant pathways [56]. Therefore, exosomes released by cancer cells and stromal cells may have a potential to modulate sensitivity of cancer cells to distinct therapies.

#### Tumor immune escape

Initially reported as tumor-associated antigens and tumor immune response stimulators, the recent studies have shown that tumor-derived exosomes might rather perform immunosuppressive functions. Tumor exosomes block the differentiation of murine myeloid precursor cells into dendritic cells (DC) [57]. Tumor exosome-carried TGF-β1 skews IL-2 responsiveness in favor of regulatory T cells and away from cytotoxic cells [58]. Human nasopharyngeal carcinoma-derived exosomes recruit, expand, and regulate the function of regulatory T cells through CCL20 [59]. NPC cell-derived exosomes impair T cell function, which is associated with upregulated miRNAs in the exosomes [60]. Tumor cell-derived exosomes switch the differentiation of myeloid cells to myeloid-derived suppressor cells (MDSCs) and induce accelerated lung metastasis in a MyD88-dependent manner [61, 62]. Hsp72 on tumor-derived exosomes promotes the immunosuppressive activity of MDSCs via autocrine activation of IL-6/STAT3 pathway [63]. Breast cancer cell-derived exosomes simulate the activation of NF-κB and enhance the secretion of pro-inflammatory cytokines in macrophages [64]. Exosomes from human prostate cancer cells express ligands for NKG2D on their surface and downregulate NKG2D expression on natural killer (NK) and CD8+ T cells, leading to the impairment of their cytotoxic function [65]. Collectively, these data suggest that tumor-derived exosomes interfere on multiple levels with the immune system to drive tumor immune evasion.

#### Tumor-stroma interaction

Tumor stroma is believed to be critically involved in tumor development and progression. Webber et al*.* suggest that prostate cancer cells could trigger differentiation of fibroblasts into myofibroblasts through exosomal TGF-β [66]. In addition, prostate cancer exosomes triggered TGFβ1-dependent fibroblast differentiation resemble stromal cells isolated from cancerous prostate tissue [67], which accelerates tumor growth by supporting angiogenesis. MSCs function as precursors for tumor myofibroblast. The research from our lab suggests that tumor cell-derived exosomes could induce differentiation of human MSCs to carcinoma-associated fibroblasts (CAFs) [68]. Adipose tissue-derived MSCs treated with breast cancer-derived exosomes also display the characteristics of myofibroblasts [69]. Moreover, stromal communication with cancer cells modulates therapy response. Boelens et al. suggest that exosomes transferred from stromal cells to breast cancer cells constitute a juxtacrine NOTCH3 pathway to expand therapy-resistant tumor-initiating cells [70]. Luga et al. demonstrate that fibroblast-secreted exosomes mobilize autocrine Wnt-planar cell polarity (PCP) signaling to drive breast cancer cell invasion and metastasis [42]. Therefore, exosomes may mediate a reciprocal interplay between tumor cells and stromal cells to synergistically promote tumor progression.

#### Tumor thrombosis

Tissue factor (TF) overexpression is closely associated with tumor progression. TF can get incorporated into tumor-derived exosomes. The hypercoagulable state in cancer patients may be partially influenced by the release of TF-bearing exosomes from tumor cells. Garnier et al. demonstrate that exosomes link the procoagulant status with metastatic phenotype in cancer. Induction of EMT changes in epithelial cancer cells results in the release of exosomes containing elevated level of tissue factor. Importantly, TF-rich exosomes can be transferred to endothelial cells and cause their exaggerated procoagulant conversion [71], suggesting that EMT influences tumor-vascular interaction through altered TF-containing exosomes. However, the exact roles of exosomes in tumor thrombosis and consequent impact on tumor growth, progression, and metastasis remain to be further explored.

### Exosomes as cancer biomarkers and targets

The findings that exosomes play critical roles in almost all aspects of cancer provide opportunities for the development of exosomes as ideal diagnostic biomarkers and therapeutic targets. Exosome-shuttled proteins and nucleic acids have been suggested as novel diagnostic and prognostic indicators for a variety of cancers. Moreover, utilizing tumor-derived exosomes as vaccines and exosomes from distinct sources as carriers for drugs and small molecules have been proved to be effective in pre-clinical studies and clinical trials.

### Exosomes as cancer diagnostic biomarkers

Exosomes are readily accessible in nearly all body fluids including blood, urine, saliva, and ascites. Exosomes contain bioactive molecules that reflect the pathological state of the originated cells, thus providing an enriched source of biomarkers (Table 2). The level of exosomes is elevated in the plasma of some cancer patients as compared to healthy controls. There is a positive correlation between the abundance of tumor exosomes and tumor stage in ovarian cancer patients [72]. Tumor is characterized by a specific miRNA profile. The majority of circulating microRNAs is concentrated in exosomes [73]. Exosomal miRNAs have been suggested as diagnostic and prognostic indicators for lung cancer, esophageal squamous cell carcinoma, prostate cancer, breast cancer, glioblastoma, ovarian cancer, and other cancer types [74–80]. Exosomal miRNAs are positively correlated with the stage and degree of cancer progression. In addition to miRNAs, long non-coding RNAs (LncRNAs) are also detected in exosomes [81, 82]. LncRNA from serum of gastric cancer patients is defined as a novel exosomal biomarker [83, 84].

**Table 2:** Exosomes from distinct biofluids of cancer patients as biomarkers

Exosomal cargos	Cancer types	Methods	Clinical value	Biofluids	References
CD34	Acute myeloid leukemia (AML)	Immunoaffinity capture	Higher levels of CD34+ exosomes in AML patients	Plasma	[117]
EDIL-3/Del1	Bladder cancer	Western blot	Elevated expression in patients with high-grade bladder cancer	Urine	[118]
miR-101, 372, 373	Breast cancer	qRT-PCR	Highly expressed in breast cancer patients and elevated miR-373 expression in receptor-negative breast cancer patients	Serum	[119]
miR-21, 146a	Cervical cancer	qRT-PCR	Elevated expression in exosomes from cervical cancer patients than healthy controls and HPV(+) subjects	Cervicovaginal lavages	[120]
Let-7a, miR-1229, 1246, 150, 21, 223, 23a	Colon cancer	qRT-PCR	Highly expressed in exosomes from colon cancer patients	Serum	[121]
CD147, CD9	Colon cancer	Exoscreen	Higher levels of CD147/CD9 double-positive extracellular vesicles in cancer patients than healthy controls	Serum	[122]
miR-17-92a cluster	Colon cancer	qRT-PCR	Elevated expression in cancer patients and higher levels predict poorer prognoses	Serum	[123]
miR-21	Esophageal squamous cell carcinoma (ESCC)	qRT-PCR	Exosomal levels of miR-21 are significantly higher in patients with ESCC than those with benign diseases	Serum	[80]
LINC00152	Gastric cancer	qRT-PCR	Elevated expression levels in gastric cancer patients than healthy controls	Plasma	[83]
EGFRvIII (mRNA)	Glioblastoma	Nested RT-PCR	Mutated EGFRvIII could be detected in exosomes from 7 of 25 glioblastoma patients but not that from 30 healthy subjects	Serum	[75]
miR-718	Hepatocellular carcinoma (HCC)	qRT-PCR	Decreased expression of miR-718 in exosomes from HCC cases with recurrence after liver transplantation compared with those without recurrence	Serum	[124]
miR-21	Hepatocellular carcinoma (HCC)	qRT-PCR	Higher exosomal levels in patients with HCC than those with hepatitis or healthy controls	Serum	[125]
miR-17-3p, 21, 106a, 146, 155, 191, 192, 203, 205, 210, 212, 214	Lung cancer	miRNA array	Total exosome and miRNA levels are upregulated in lung cancer patients and these 12 miRNAs could be detected in exosomes	Plasma	[76]
LRG1	Lung cancer	Western blot	Patients with non-small cell lung cancer have an increased LRG1 expression in exosomes compared to healthy controls	Urine	[126]
TYRP2, VLA-4, Hsp70, MET	Melanoma	Western blot, multiplex protein analysis	The levels of these 4 proteins are increased in exosomes from stage III and IV patients compared to stage I patients as well as healthy controls	Plasma	[39]
CD63, caveolin-1	Melanoma	In-house sandwich ELISA (Exotest)	Melanoma patients have more CD63- and caveolin-1-positive exosomes compared to healthy controls	Plasma	[127]
Galectin-9	Nasopharyngeal carcinoma (NPC)	Western blot	Exosomes from NPC patients but not that from healthy controls contain galectin-9	Serum	[128]
Claudin-4	Ovarian cancer	Western blot	Claudin-4 could be detected in exosomes from 32 of 63 ovarian cancer patients but only 1 of 50 healthy controls	Plasma	[129]
miR-21, 141, 200a, 200b, 200c, 203, 205, 214	Ovarian cancer	miRNA array	The levels of these 8 miRNAs are elevated in exosomes from ovarian cancer patients compared to healthy controls and benign tumors	Serum	[72]
miR-1246, 4644, 3976, 4306	Pancreatic cancer	qRT-PCR	Upregulated expression in pancreatic cancer patients compared to healthy controls	Serum	[130]
PTEN	Prostate cancer	Western blot	PTEN is exclusively expressed in exosomes of prostate cancer patients compared to healthy controls	Plasma	[131]
Survivin	Prostate cancer	Western blot, ELISA	Prostate cancer patients have more survivin-positive exosomes compared to healthy controls as well as patients with benign prostatic hyperplasia	Plasma	[132]
PSA, PSMA	Prostate cancer	Western blot	Detected in 20 of 24 exosomes from prostate cancer patients but not in healthy controls	Urine	[133]
miR-1290, miR-375	Prostate cancer	qRT-PCR	Highly expressed in castration-resistant prostate cancer patients and their levels are significantly associated with poor overall survival	Plasma	[134]
LncRNA-p21	Prostate cancer	qRT-PCR	Higher level of exosomal lncRNA-p21 in patients with prostate cancer than those with benign hyperplasia	Plasma	[135]

### Exosomes as cancer therapy targets

#### Exosome-based immunotherapy

Dendritic cell-derived exosomes (dexosomes) have been developed as immunotherapeutic anticancer agents [85]. Tumor peptide-pulsed DC-derived exosomes suppress growth of established murine tumors in a T cell-dependent manner [86]. Exosomes secreted by living tumor cells contain and transfer tumor antigens to dendritic cells and induce potent CD8+ T cell-dependent antitumor effects on mouse tumors [87]. Dexosomes have entered clinical trials for colorectal cancer, metastatic melanoma, and non-small cell lung cancer and have achieved modest therapeutic effects [88].

#### Exosome removal for cancer therapy

The removal of exosomes from advanced cancer patients is a novel strategy to treat cancer [89]. Exosome depletion by dimethyl amiloride (DMA) in mice restores the anti-tumor efficacy of cyclophosphamide (CTX) through the inhibition of MDSC functions. Amiloride, a drug used to treat high blood pressure, inhibits exosome formation and blunts MDSC suppressor functions in colorectal cancer patients [63]. The biotechnology company Aethlon Medical has developed an adjunct therapeutic method HER2osome, which is able to reduce tumor-secreted HER2 positive exosomes in the circulation and thus inhibit HER2-positive breast cancer progression. However, further work is needed to evaluate the clinical safety of such a treatment strategy based on exosome removal.

#### Exosomes as anti-cancer drug delivery vehicles

The use of exosomes as nucleic acid or drug delivery vehicles has gained considerable interest due to their excellent biodistribution and biocompatibility [90]. Exosome-mediated delivery of therapeutic short interfering RNA (siRNA) to the target cells has been tested. The exosome-delivered siRNA is effective at causing post-transcriptional gene silencing and inducing cell death in recipient cancer cells [91–93]. To improve drug delivery efficacy to tumors, the researchers have modified exosomes with targeting ligands such as iRGD-Lamp2b. The modified exosomes show highly efficient targeting to αV integrin-positive breast cancer cells, and intravenous injection of these exosomes obviously inhibits tumor growth [94]. In addition, exosomes have been utilized as effective vehicle for drug delivery [95]. Exosomes from MSCs have been tested as the vehicle to package and deliver active drugs such as paclitaxel [96].

## Conclusion

The rapid expansion of the number of published studies on exosomes clearly shows that research on exosomes and their functions is now a very exciting field. Exosomes are small particles with big roles and are emerging as major players in intercellular communication. Exosomes have been suggested as active transporters for proteins, DNA, mRNA, and non-coding RNAs. The roles of exosomes in cancer have been gradually realized. Although some reports have suggested anti-tumor roles of exosomes due to their potential to elicit immune response, most of the reports have revealed the various pro-tumor effects of exosomes, which is further supported by the observations that the level of circulating exosomes is increased in cancer patients and correlated with tumor progression. In this review, we discussed several aspects of exosome biology in cancer. Cancer cells communicate with the surrounding and distant cells via exosomes, which constitutes a bi-directional interaction network to synergistically promote cancer development, progression, metastasis, and drug resistance. However, the exact mechanisms mediating the complex roles of exosomes in cancer have not yet fully elucidated. Exosomes would be ideal biomarkers for cancer diagnosis and targeted therapy because they closely represent the state of their parental cells and are relatively stable in the circulation and could be easily collected from body fluids. The potential of exosomal contents for diagnostic and prognostic biomarkers have been investigated in various cancers. It is required to develop faster and more convenient methods for validating the proposed exosomal cargos as biomarkers in specimens from human cancer patients. The use of nanotechnology to load exosomes with small molecules or drugs for cancer therapy has also been exploited. Improvements in developing new strategies to obtain a large amount of exosomes from appropriate donor cells, efficiently introducing the therapeutic agents into exosomes, and optimizing the targeted delivery of exosomes to particular tissues will facilitate the use of exosomes as natural carrier in clinical therapy. Future studies of exosomes will not only shed lights on their roles in the pathogenesis of cancer but will open new avenues for cancer diagnosis and therapeutics.

## Abbreviations

APC: antigen-presenting cell
ASCs: adipose-derived stem cells
BM-MSCs: bone marrow mesenchymal stromal cells
CAFs: carcinoma-associated fibroblasts
CTX: cyclophosphamide
Dexosomes: dendritic cell-derived exosomes
DMA: dimethyl amiloride
EMT: epithelial-mesenchymal transition
ESCRT: endosomal sorting complex required for transport
esRNA: exosomal shuttle RNA
FIH-1: factor-inhibiting hypoxia-inducible factor 1
GTPases: guanosine triphosphatases
HIF-1: hypoxia-inducible factor 1
HSPGs: heparan sulfate proteoglycans
ICAM: intercellular adhesion molecule
ILVs: Intraluminal vesicles
LncRNA: long non-coding RNA
MACS: magnetic activated cell sorting
MDR: multi-drug resistance
MDSCs: myeloid-derived suppressor cells
MVBs: multivesicular bodies
NPC: nasopharyngeal carcinoma
PCP: planar cell polarity
RISC: RNA-induced silencing complex
siRNA: short interfering RNA
SP: side population
TEM: transmission electron microscopy
TF: tissue factor
TIM: T cell immunoglobulin and mucin domain molecule
TLR: toll-like receptor
TSAP6: tumor suppressor-activated pathway 6
